# The Roles of Exosomes in Ovarian Cancer Chemo-resistance

**DOI:** 10.7150/jca.84930

**Published:** 2023-07-09

**Authors:** Yang Liu, Xiao Li, Tianyu Zhang, Guoyan Liu

**Affiliations:** 1 Departments of Obstetrics and Gynecology, Tianjin Medical University General Hospital, Tianjin, 300052, China.; 2 Departments of Obstetrics and Gynecology, Tianjin Medical University General Hospital, Tianjin, 300052, China.; 3 Department of Gynecologic Oncology, Tianjin Medical University Cancer Institute and Hospital, Huanhuxi Road, Hexi District, Tianjin, 300060, China.

**Keywords:** Exosomes, Ovarian cancer, Chemotherapy resistance, Therapeutic potential

## Abstract

As common gynecological oncology, ovarian cancer has a high fatality rate and poor overall survival, mainly because of nonspecific symptoms in the early stages and chemotherapy resistance. Exosomes, nano-sized vesicles secreted by almost all types of cells, carry valuable commodities such as proteins, lipids, enzymes, mRNAs, and miRNAs between cells. They take part in remodeling the tumor microenvironment, promoting tumor angiogenesis and metastasis, and regulating immune metastasis and chemotherapy resistance in ovarian cancer. Previous studies have reported that exosomes could transfer chemotherapy resistance from drug-resistant tumor cells to sensitive ones by delivering proteins and miRNAs. Also, exosomes are involved in chemotherapy resistance by transferring multidrug-resistance-related transporters, decreasing apoptosis, promoting epithelial-to-mesenchymal transition, and changing signal transduction pathways. Furthermore, they play a significant role in early detection, chemotherapy efficacy evaluation, and treatment of ovarian cancer. Exosomes are applied as chemotherapeutic delivery vehicles and therapeutic targets to inhibit anti-tumor immune responses. In addition, exosomes can be developed for cancer immunotherapy because of their immunomodulatory potential. Therefore, the article reviews the latest research progress of exosomes in ovarian cancer to elaborate on the mechanisms of exosome-mediated chemotherapy resistance in ovarian cancer patients and provide a forecast on their clinical therapeutic potential in improving chemotherapy sensitivity.

## Introduction

Ovarian cancer is the most lethal gynecologic malignancy among female patients and the eighth cause of cancer-related deaths in women worldwide. There were approximately 19,710 estimated new cases and 13,270 estimated deaths of ovarian cancer, accounting for 5% of all cancer deaths in the United States in 2023^1^. The standard treatment for advanced ovarian cancer patients includes cytoreductive surgery to remove tumor tissues as much as possible, combining platinum-based chemotherapy. However, ovarian cancer patients are generally diagnosed at a late stage. The five-year relative survival rate from 2012 to 2018 remains only about 50% due to the lack of effective treatment strategies in recurrent ovarian cancer patients^1^. Although the initial response to platinum-based chemotherapy is good, most ovarian cancer patients confront the problems of high recurrence risk and poor survival terms because of resistance to chemotherapy. Therefore, exploring the molecular mechanisms of chemotherapy failure, tumor invasion, and metastasis in ovarian cancer patients has been a clinical challenge. The mechanisms of chemotherapeutic resistance include a variety of aspects such as increased DNA damage repair, drug efflux and influx, drug inactivation and alteration of drug targets, inactivation of apoptosis pathways, activation of epithelial-to-mesenchymal transition (EMT) pathways, and miRNAs-mediated mechanisms. In recent years, it has been widely known that exosomes are important in impacting the progression and metastasis of ovarian cancer by mediating EMT, programming non-coding RNAs like miRNAs, angiogenesis, immune suppression, and forming a pre-metastatic niche^2^. Furthermore, it could be a novel biomarker and therapeutic target for treating ovarian cancer patients owing to its accessibility, circulation stability, and high specificity. Therefore, this review aims to explain the roles of exosomes in chemotherapy resistance and attempt to seek the therapeutic potential of exosomes in improving the prognosis for ovarian cancer patients.

## Biogenesis, composition, and function of exosomes in ovarian cancer

**Figure 1:**
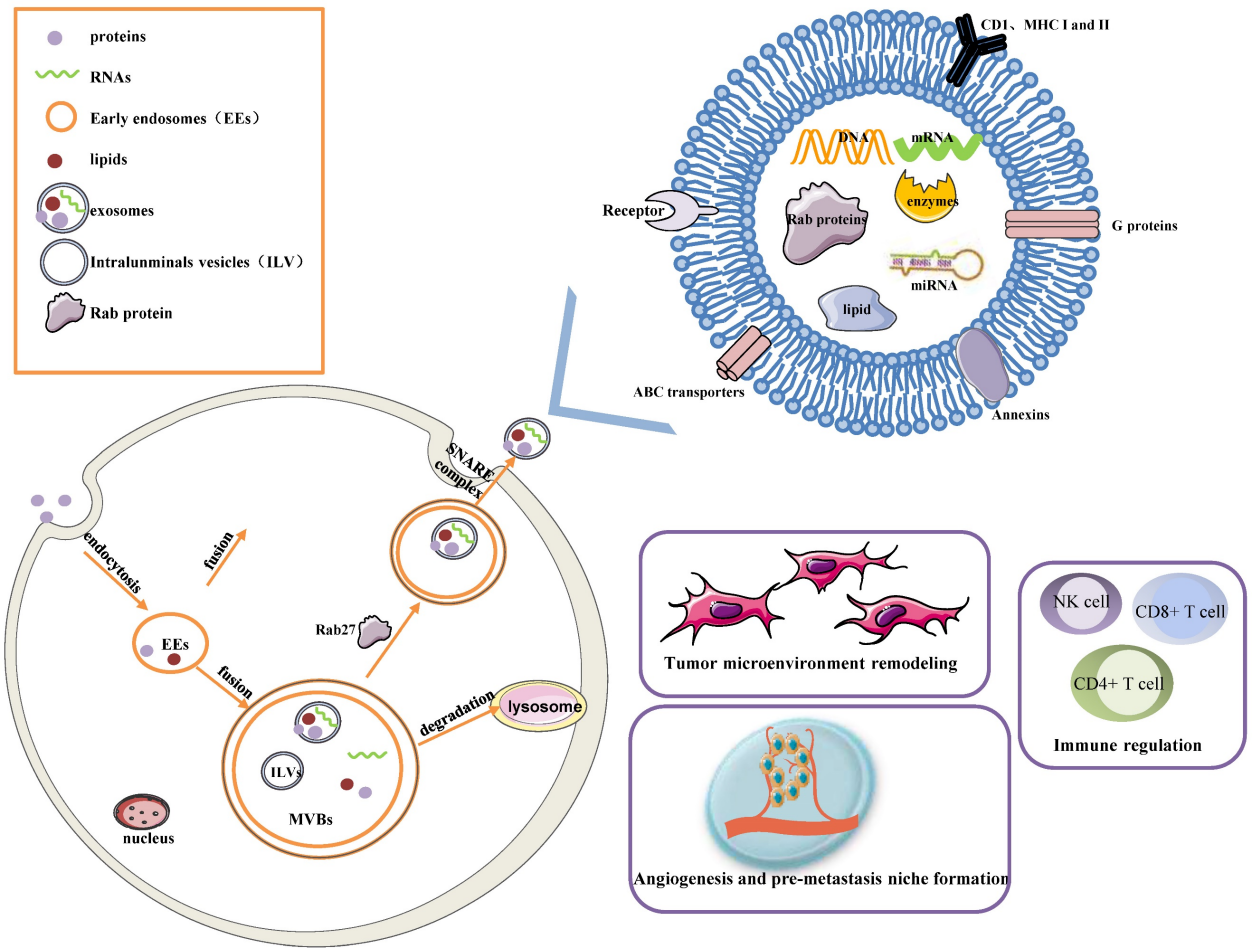
The biogenesis, Structure, and functions of the exosome. Exosomes are 50-100 nm nanoscale vesicles with a lipid bilayer membrane. They can carry many essential molecules like proteins, mRNA, miRNA, lipids, and enzymes. Distinct proteins are shown in the figure, such as CD1, MHC I and II, Rab proteins, Annexins, ABC transporters, and signaling transduction-related proteins. Early endosomes are formed by the fusion of primary endocytic vesicles. MVBs fuse with the plasma membrane through the SNARE complex and release their contents called exosomes from the ILVs into the extracellular space. The contents of exosomes can be transferred from the cells to target cells in a local environment or at a distant site in the tumor environment. Exosomes greatly influence a few pathways, such as tumor microenvironment remodeling, signal transduction, tumor angiogenesis and metastasis, immune regulation, and chemotherapy resistance.

Early endosomes are formed by the fusion of primary endocytic vesicles. Then Multivesicular bodies (MVBs) information begins by inward membrane budding, leading to cargo sequestration and distribution into vesicles. MVBs, also called late endosomes to escape degrading lysosomes in the presence of a high concentration of ceramide lipid family, fuse with the plasma membrane through soluble N-ethylmaleimide (NEM)-sensitive factor attachment protein receptor (SNARE) complex and release their contents called exosomes from the intraluminal vesicles (ILVs) into the extracellular space. In addition, Rab27A and Rab27B are the essential mediators to lead the MVBs toward the cell periphery^3^. Exosomes are 50-100 nm nanoscale vesicles with a typical lipid bilayer membrane structure without cellular organelles. They promote intercellular communication by transferring contents of their tumor-initiating cells, such as proteins, mRNA, miRNA, lncRNAs, lipids, and nucleic acids^2^. For example, exosomes contain many proteins such as the major histocompatibility complex (MHC) class I and II in presenting antigen^4^, Annexins and Rab proteins in regulating membrane cytoskeleton dynamics and membrane fusion^5^, signaling transduction-related proteins like G proteins^6^. In addition, exosome lipids could not only take part in exosome biogenesis and maintain exosome shape but also regulate the homeostasis in the recipient cells by changing the lipid composition, especially in cholesterol and sphingomyelin. miRNAs derived from exosomes could participate in many processes like oncogenesis, pre-metastatic niche formation, tumor metastasis and invasiveness, as well as drug resistance. miRNAs from exosomes could transmit drug resistance and change drug sensitivity in the recipient cells through modulating cell cycle distribution and apoptosis. Fig. 1 describes the biogenesis, structure, and function of exosomes.

Exosomes greatly influence a few pathways, such as tumor microenvironment remodeling, signal transduction, tumor metastasis and invasion, immune regulation (antigen presentation, immune activation, and suppression), and chemotherapy resistance, by releasing bioactive molecules^7^, ^8^. The roles of each exosome vary depending on their cellular origin. Exosomes are secreted by many types of cells including lymphocytes, dendritic cells, macrophages, stem cells, epithelial cells, endothelial cells, and tumor cells^9^. They keep functional until entering or fusing with a recipient cell in local surroundings or distant regions, so it is an essential method of intercellular communication to transfer exosomes in the tumor microenvironment. Furthermore, molecules released from exosomes are served as diagnostic tools for the early detection of ovarian cancer and prognostic biomarkers in ovarian cancer patients.

Meanwhile, tumor-derived exosomes modulated the tumor microenvironment which consisted of tumor cells, stromal cells, mesenchymal cells, infiltrating immune cells, and cytokines by stimulating extracellular receptor signaling. Ovarian cancer-derived exosomes induced fibroblasts to cancer-associated fibroblasts (CAFs) and promoted the production of transforming growth factor 1 (TGFβ1) and activation of somatic mutations in the Mothers Against Decapentaplegic Homolog (SMAD) signaling. In the hypoxic microenvironment, it suggested that tumor cells secreted more exosomes with enhanced angiogenic and metastatic potential to promote tumor microenvironment alteration and tumor progression^10^. It has been reported that the P53 pathway increases the number of exosomes and rate of exosomes production under stress like hypoxia and toxic through regulating transcription of the various genes such as Tumor suppressed activated pathway6 (TSAP6), a P53-regulated gene product^11^. In hypoxia conditions, tumor-derived extracellular vesicles induced metabolic change of glycolytic pathway proteins, promoted resistance to carboplatin, and facilitated tumor progression^12^.

Exosomes released by tumor cells carried vascular endothelial growth factor (VEGF), transforming growth factor β (TGF-β), and tumor necrosis factor α (TNF-α) to induce vascular formation which is important for tumor growth and metastasis. Growing evidence on exosomes' function in angiogenesis among different types of tumors indicated that tumor-derived exosomes were essential tools for promoting angiogenesis. Epithelial ovarian cancer cells derived exosomes could transfer metastasis-associated lung adenocarcinoma transcript 1 (MALAT1) to recipient HUVECs and stimulate angiogenesis-related gene expression in recipient human umbilical vein endothelial cells (HUVECs), eventually promoting angiogenesis^13^. Soluble E-cadherin in exosomes, an activator of angiogenesis within the pre-metastatic microenvironment could bind with VE-cadherin on endothelial cells, activate a signaling cascade, ultimately activate β-catenin and NF-κB which stimulate endothelial cell migration and overall vascular permeability^14^. In 2020, Dehghi et al observed that ovarian cancer cell-derived exosome transferred miR-141-3p to endothelial cells and up-regulated the JAK-STAT3 pathway by decreasing the expression of cytokine-inducible suppressors of cytokine signaling (SOCS)-5. Furthermore, they also found that miR-141-3p could promote endothelial cell angiogenesis and metastasis by up-regulating the expression of VEGFR-2^15^.

Exosomes derived from immune cells play an important role in regulating immune response. For example, exosomes derived from antigen-presenting cells (APCs) are enriched in antigen-presenting molecules (MHC-I and MHC-II) on their surface and present antigens to T cells and activate the immune response^16^. Tumor-derived exosomes could play a dual role in maintaining immune homeostasis, either activating immune response or activating immunosuppressive changes. TD-exosomes could inhibit the immune response by inducing the apoptosis of effective T lymphocytes by the Fas/FasL pathway, downregulate the activity of NK cells via NKG2D ligands, and inhibit CD4+ and CD8+ T cells proliferation by decreasing antigen-presenting cells^4^, ^9^. On the other hand, TD-exosomes delivered tumor-associated antigen (TAA) to other APCs, which could activate naïve CD4+T cells in vivo. Similarly, M1 macrophage could increase the release of proinflammatory cytokines, promote T cell-mediated immune response and mediate TD-derived exosomes to establish a local immunostimulatory microenvironment^17^. However, it remains unclear how TD-EVs deliver multiple signals to regulate the dual immune response in immune cells.

## Primary and acquired chemo-resistance in ovarian cancer

The most common ovarian tumor is epithelial carcinoma, classified into seven main histological subtypes: high-grade and low-grade serous, mucinous, endometrioid, clear cell, Brenner, seromucous, and undifferentiated carcinomas, according to the World Health Organization. One of the epithelial carcinomas is high-grade serous ovarian carcinoma accounting for 70%, of which the majority derives from the fallopian tube epithelium and leads to the most significant ovarian cancer deaths. About 40-50% of high-grade serous ovarian cancer shows homologous recombination (HR) deficiency caused by germline or somatic mutation of breast cancer susceptibility gene (BRCA1/2) and other DNA repair genes or epigenetic silencing through methylation. Moreover, it shows high chromosome instability and ubiquitous TP53 mutations^18^. BRCA mutations increased the risk of epithelial ovarian cancer (EOC) and were associated with improved 5-year overall survival, with BRCA2 carriers having the best prognosis (52%) in invasive EOC^19^.

The response rate to initial first-line chemotherapy drugs for patients diagnosed with high-grade serous ovarian cancer (HGSOC) is greater than 80%, but the 5-year survival rate for them is between 35% and 40%^20^. Recently, it has become an efficient strategy to target HR-deficient cancers. Because of their synthetic lethality, Poly (ADP-ribose) polymerases (PARP) inhibitors attract significant attention in treating recurrent ovarian cancers with HR repair defects. In a randomized, open-label, phase II study, Olaparib plus paclitaxel and carboplatin followed by maintenance Olaparib monotherapy significantly improved progression-free survival (PFS) versus paclitaxel plus carboplatin alone, with the most significant benefit in BRCA-mutated patients, and had an acceptable and manageable tolerability profile^21^.

Although platinum-based chemotherapy is currently considered the primary therapy for high-grade serous ovarian cancer patients, chemotherapy resistance remains significant obstruction in treating ovarian cancer patients. In addition, the issue of PARPi resistance has emerged in clinical cancers, and the mechanisms of PARPi-acquired resistance remain to be investigated. Therefore, it is essential to elucidate the molecular mechanisms in chemotherapeutic resistance and design novel and effective therapeutic agents targeting resistant cancer cells to improve survival in relapsed HGSOC patients.

However, molecular mechanisms associated with platinum-intrinsic resistance still need to be fully understood. CCNE1(Cyclin E) amplification which encodes cyclin E1, not only provides an oncogenetic stimulus through activation of the cell cycle to promote the occurrence of malignant tumors; but increases platinum resistance and recurrence rate because of chromosome instability, ultimately leading to poor prognosis of HGSOC patients^22^. Dariush et al. proved that siRNA-mediated knockdown of CCNE1 resulted in G1/S phase arrest and reduced cell viability and apoptosis. They decreased the formation of clonogenic cells after cisplatin treatment in 19q12-amplified OVCAR-3 cell lines^23^. They observed that CCNE1 amplificated in 20% of high-grade serous ovarian cancer with the guide of the Cancer Genome Atlas project^18^. In addition, high-level amplification and over-expression of CCNE1 identify ovarian cancer patients with a sufficiently poor prognosis^24^. So CCNE1 amplification is the dominant structural variant associated with primary chemotherapy failure. Another analysis by Dariush et al. identified two mechanisms associated with primary resistance in advanced serous ovarian cancer, involving amplifying 19q12, which contains CCNE1 related to TPX2 amplification and 20q11.22-q13.12 and mapping immediately adjacent to the steroid receptor coactivator NCOA3. The discovery was completed in a clinically well-characterized sample population of 118 ovarian cancers using high-resolution oligonucleotide microarrays. This implied that CCNE1 amplification and enhanced extracellular matrix deposition had clinical relevance for chemotherapy response prediction^25^.

**Figure 2:**
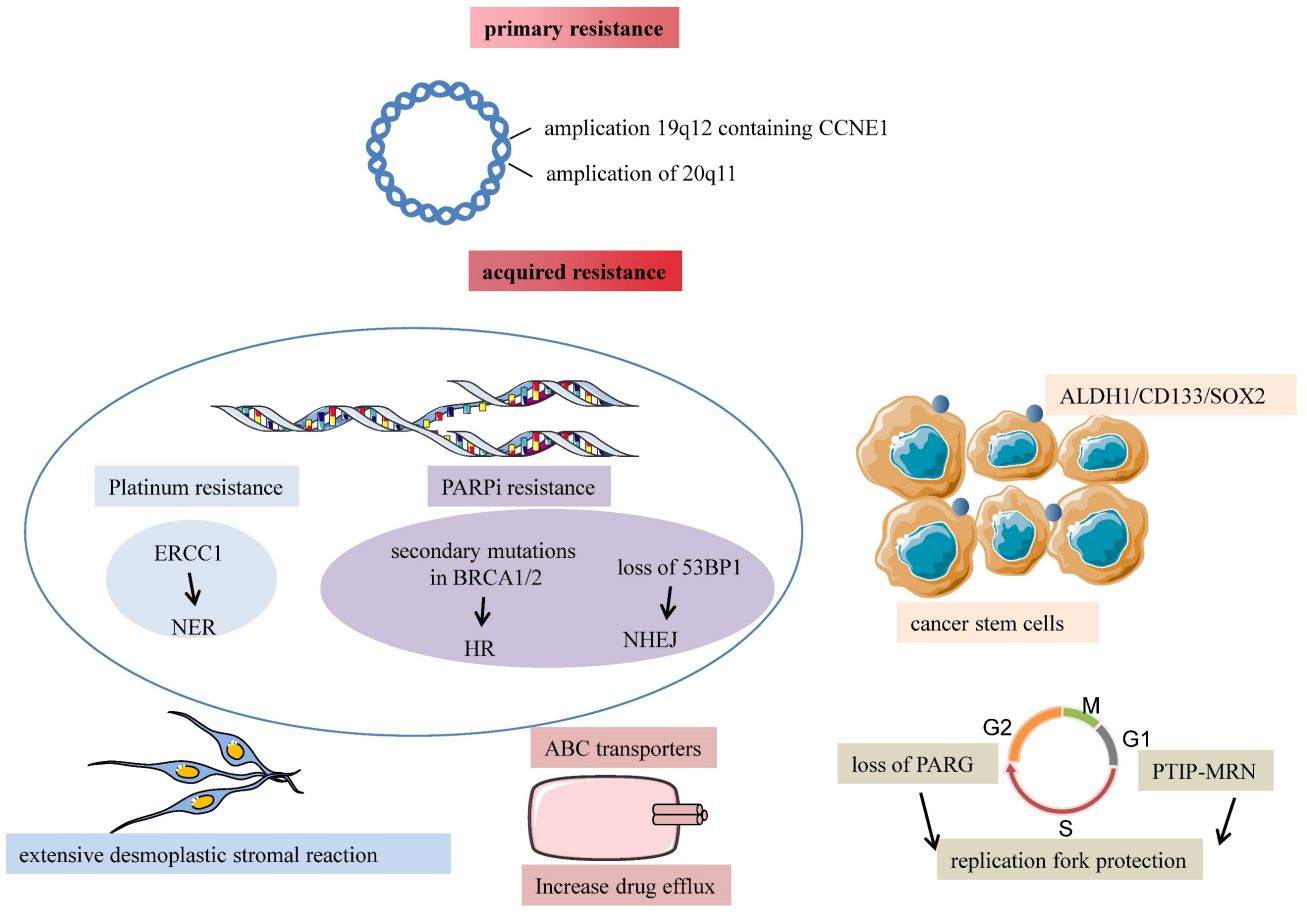
Major mechanisms of chemotherapy resistance in ovarian cancer are divided into primary and acquired resistance. Amplification of 19q12 contains CCNE1 related to TPX2 amplification, and 20q11.22-q13 are two dominant structural variants associated with primary chemotherapy failure. Tumor desmoplasia reaction is associated with poor response to chemotherapy despite a homologous-recombination defection. CSCs markers ALDH1, CD133, and SOX2 are associated with non-response to chemotherapy. Platinum resistance is attributed to enhanced NER because of the increased capacity of the cells to repair platinum-induced DNA damage. Essential mechanisms of acquired PARPi resistance are restoring the homologous recombination repair because of secondary mutations of RCA1/2, loss of 53BP1 resulting in HR reactivation, and alterations in the PARP and the PARG proteins leading to the same replication fork protection.

Almost 75% of patients sensitive to initial chemotherapeutic compounds relapse within two years because of acquired resistance. Ovarian cancer is a disease characterized by extensive structural genomic variation. Patch et al. used whole-genome sequencing of tumor and germline DNA samples from 92 patients with primary refractory, resistant, sensitive, and matched acquired resistant disease. They revealed that gene breakage commonly inactivated the tumor suppressor genes RB1, NF1, RAD51B, and PTEN in HGSOC and substantially contributed to acquired chemotherapy resistance. As known, chemotherapy resistance is a complicated phenomenon consisting of many mechanisms, including drug inactivation, drug target alteration, upregulation of drugs efflux bumps, DNA damage repair mechanisms, apoptosis inhibition, EMT, epigenetic effects like miRNAs, modifications of the tumor microenvironment, cancer stem cells, or any combination of these mechanisms 26. We discuss three main mechanisms of acquired resistance in the following paragraphs (shown in Fig. 2). The rest will be described in the third chapter, “Exosome-mediated chemotherapy resistance in ovarian cancer”.

Ovarian cancer is a peritoneal disease where tumor tissues spread directly to surrounding organs and implant on the surface of the omentum and abdominal organs. The omentum is a highly vascularized connective tissue with many cells like adipocytes, fibroblasts, lymphocytes, and macrophages. Au Yeung suggested that miR21-containing exosomes released by cancer-associated adipocytes (CAAs) or cancer-associated fibroblasts (CAFs) within the omental microenvironment significantly promoted the acquisition of a platinum-resistant phenotype in ovarian cancer^27^. Patch et al. supported that recurrent HGSOC showed an extensive desmoplastic stromal reaction, and it's possible that tumor desmoplasia reaction was associated with tumor recurrence and poor response to chemotherapy despite a homologous-recombination defection^28^. So tumor microenvironment and omentum structure influence tumor development and chemotherapy response.

Many studies illustrate that ovarian cancer cells expressing stemness markers cause tumor chemotherapy resistance and promote tumor development in ovarian cancer patients. Cancer stem cells are defined as a subpopulation of cancer cells with the potential for self-renewal, infinite proliferation, and multiple differentials. The following surface and intracellular markers have been discovered in ovarian cancer stem cells: aldehyde-dehydrogenase (ALDH), CD133, CD44, CD177, CD24, and EpCAM^29^. It's been proposed that subpopulations of tumor cells with stem cell-like properties maintain the growth of resistant tumor cells and lead to tumor recurrence because side populations (SP) overexpress ATP binding Cassette (ABC) drug transporters. Rizzo et al. observed that ascites from relapsed patients had more ovarian cancer SP cells than chemo-sensitive patients, ABCB1 and EZH2 were overexpressed in SP compared with non-SP patients' tumor cells^30^.

Chemotherapy-resistant marker like ALDH1 exists on the surface of cancer stem cells and plays important roles in drug resistance. They found that the overexpression of ALDH1 was observed in paclitaxel- and topotecan-resistant cell lines, indicating that ALDH1-positive cancer cells were related to drug resistance and tumor development in ovarian cancer^31^. Chen found that expression of CSCs markers CD133 and SOX2 were associated with non-response to chemotherapy and shorter overall survival periods or disease-free survival durations in HGSOC patients^32^. Studies by Steg et al. showed that tumors collected from recurrent platinum-resistant patients had a significantly improved CD133 expression, suggesting stem cell subpopulations could ultimately contribute to tumor resistance and recurrent disease 33.

The ability of ovarian cancer cells to repair platinum-induced DNA damage can decide the efficiency of chemotherapeutic drugs. The nucleotide excision repair (NER) pathway recognizes DNA damage caused by platinum compounds, and then Excision Repair Cross-Complementing 1(ERCC1) protein, NER protein complex, repairs the lesion^34^. Selvakumaran et al. showed that ovarian cancer cells OVCAR10 resistant to cisplatin compounds increased sensitivity after silencing the ERCC1 expression using antisense RNA methodologies, demonstrating an inverse association between NER and response to platinum compounds. It suggested it could enhance cisplatin cytotoxicity by inhibiting the NER pathway in cisplatin-resistant cells^35^. Platinum resistance has been attributed to improved NER because of the increased capacity of the cells to repair platinum-induced DNA damage.

Restoring the homologous recombination repair is another important mechanism. The BRCA1/2 gene is an important section in the HR pathway; germline or somatic mutations in BRCA genes result in genomic instability and ovarian cancer development. Many studies have shown that ovarian cancer with BRCA gene mutations is more sensitive to platinum and PARP inhibitors than BRCA wild-the type tumors. Furthermore, they observed several molecular events associated with acquired resistance in recurrent samples, including secondary mutations in BRCA1/2 that restored the open reading frame, which leads to functional homologous recombination repair, loss of BRCA1 promoter somatic methylation, an molecular subtype alteration, promoter fusion, and translocation involving the 5' end of ABCB1^28^. Therefore, BRCA mutation status in the DNA repair pathway can predict the value of platinum and PARPi sensitivity in HGSOC.

Non-homologous end joining (NHEJ) is the second important DNA double-strand break (DSB) repair pathway. 53BP1(TP53BP1) promotes DSB repair by NEHJ in G1phase cells by forming 53BP1-RIF1 complexes which protect DSB ends from exonuclease processing. Loss of 53BP1 results in HR reactivation in homologous recombination deficiency tumors, rendering tumor cells resistant to PARPi^36^.

Recent studies have revealed other mechanisms to mediate PARPi resistance, including replication fork protection, increased drug efflux pumps, and alterations in the PARP and the PAR glycohydrolase (PARG) proteins^37^. Gogola et al. showed that loss of PARG increased resistance to BRCA2-deficient tumor cells by restoring PAR formation, rescuing downstream PARP1 signaling, and slowing DNA replication fork progression. Pax2 transactivation domain-interacting protein's (PTIP) absence reduces the recruitment of MRE11 nuclease (MRN) to stalled replication forks, which protects these replication forks from extensive degradation and ultimately confers resistance to PARPi. In addition, PARP1 and CHD4 lead to the same replication fork protection and acquired PARPi resistance^38^.

## Exosome-mediated chemotherapy resistance in ovarian cancer

**Figure 3:**
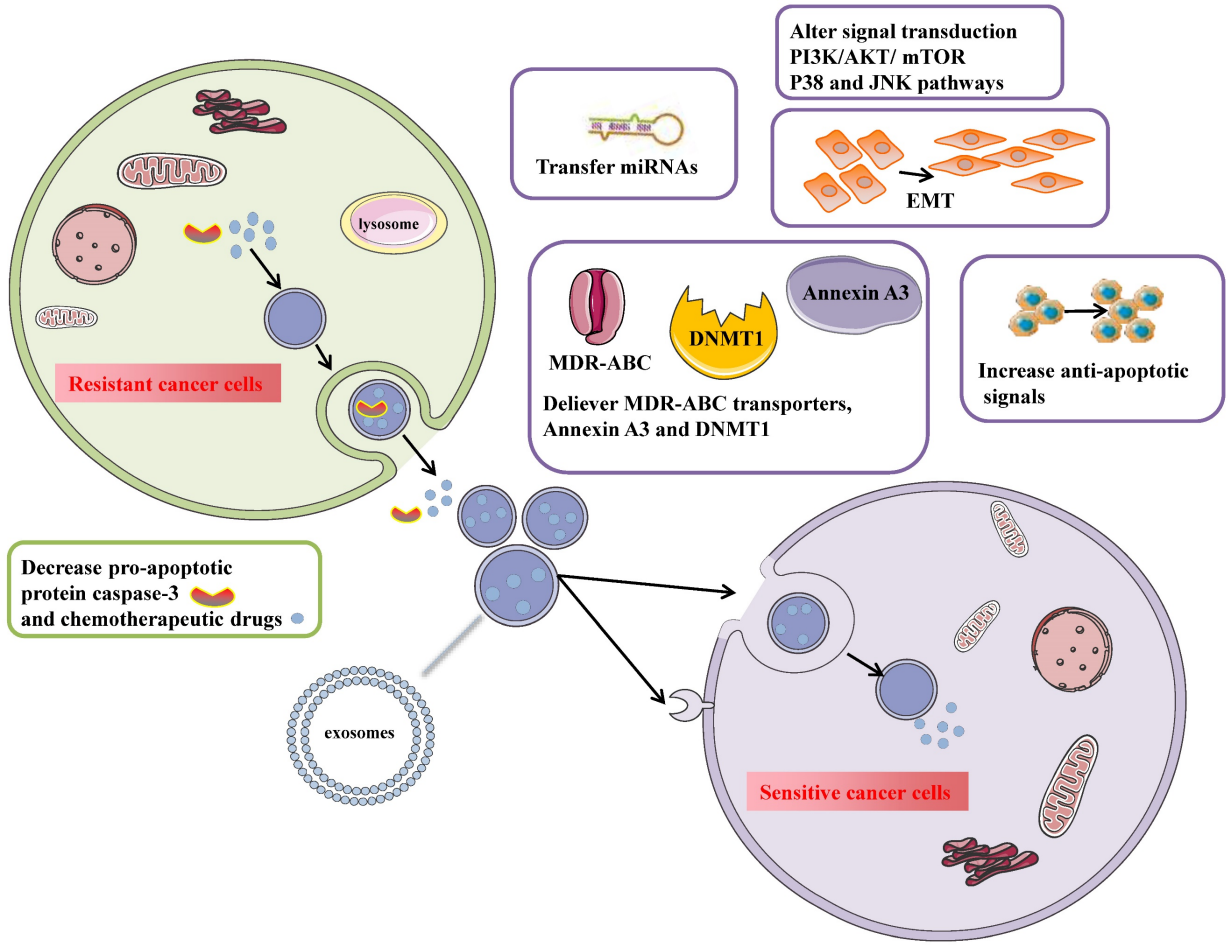
Exosomes mediate chemotherapy resistance in the donor (resistant) and the recipient (sensitive) ovarian cancer cells. In resistant/donor cells: 1) Chemotherapeutic drugs are secreted into exosomes and escape lysosome degradation. Cisplatin efflux mediated by exosomes increases, especially under hypoxic conditions. 2) Reduce the intracellular concentration of caspase3 to prevent apoptosis. 2) In sensitive/recipient cells: 1) Exosomes can transport MDR-ABC transporters (P-gp) to recipient cells to reduce intracellular drug concentration. 2) Up-regulation of Annexin A3 inhibits apoptosis and increases platinum resistance. 3) Exosomes regulate epigenetic changes by packaging DNMT1. 4) Exosomes can activate anti-apoptotic pathways through exosomes' surface-expressed receptors and transfer transcriptional factors to the recipient cells. 5) promote EMT. 6) exosomes can transfer miRNAs to recipient cells to increase chemotherapy resistance. 7)Alter signal transduction PI3K/AKT/mTOR and P38 and JNK pathways.

Tumor-derived exosomes participate in numerous pathological processes such as cancer proliferation and metastasis, immune suppression, and chemotherapy resistance. Chemotherapy resistance is divided into intrinsic resistance due to genetic or phenotypic changes and extrinsic resistance interacting with the tumor microenvironment. Exosomes transfer resistant phenotypes to drug-sensitive cells and make them resistant to drugs by delivering ABC transporters, increasing anti-apoptotic signaling, mediating EMT, and increasing tumor-stroma interaction. Except for proteins contained in exosomes, non-coding RNAs such as lncRNAs and miRNAs are loaded in exosomes and alter protein expression in recipient cells to activate chemo-resistant pathways. So the identified molecular mechanisms of exosome-mediated chemo-resistance mainly include several aspects (Fig. 3): 1) exporting drugs out of cells via exosomes; 2) drug transporters; 3) transferring annexin A3 between cancer cells^5^; 4) mediating chemotherapy resistance by increasing DNMT1^39^; 5) regulate apoptosis; 6) promote EMT^40^; 7) transfer miRNAs to sensitive cancer cells^27^, ^41^, ^42^; 8) alter transduction signals.

### Direct drug export by exosomes

Decreasing cellular anti-cancer drugs' concentration is a major mechanism in therapy resistance in ovarian cancer. The efflux of drugs and their decomposed products via exosomes reduces effective drugs' concentration and promotes drug resistance in tumor-donor cells. The hydrophobic character of drugs interacts with lipids with exosome membranes, so chemotherapeutic drugs are secreted into exosomes, contributing to drug resistance. So it is crucial to explore in-depth mechanisms of exosome-mediated cisplatin efflux and seek novel clinical therapies in chemo-resistant ovarian cancer. Safaei et al. found that the amount of cisplatin in exosomes released from cisplatin-resistant ovarian cancer cell line 2008/C13*5.25 was 2.6 times higher than that released from cisplatin-sensitive cells^43^. Cisplatin is sequestered into lysosomes whose abnormalities are found in drug-resistant cells, including aberrant morphology and degradative/secretory phenotypes. Aberrant lysosomal H+-bump is also noticed in cisplatin-resistant cells. They concluded that defective endosomal/lysosomal acidification was partly responsible for acquired cisplatin resistance because of reduced uptake^44^. A study by Safaei et al. has shown that the lysosomal compartment in cisplatin-resistant cells was reduced to 40% compared with cisplatin-sensitive cells. In addition, lysosome-associated proteins 1 and 2 (LAMP1 and LAMP2) were also reduced, and cisplatin-resistant cells released more exosomes containing more LAMP1 than cisplatin-sensitive cells. Dorayappan et al. observed that exosomes isolated from patient-derived ascites ovarian cancer cells cultured under hypoxic conditions were capable of chemo-resistance by a drug-efflux mechanism in vitro and vivo. STAT3 regulates Rab7 and Rab27a under hypoxic conditions and increases exosome release in ovarian cancer cells by promoting a more secretory lysosomal phenotype. Furthermore, cisplatin efflux via exosomes increased in ovarian cancer cells under hypoxic conditions^45^.

O-GlcNAcylation, a critical protein post-translational modification, plays a vital role in biological processes. Previous studies have reported that O-GlcNAcylation transferase (OGT) downregulation increased cisplatin resistance in ovarian cancer cells. In this study, downregulation of OGT-mediated O-GlcNAcylation of SNAP-23 promoted the formation of SNAP-23-Stx4-VAMP8 complex and increased exosomes release in ovarian cancer, which increased cisplatin efflux through exosomes and reduced the intracellular cisplatin concentration^46^.

Transmembrane family proteins (TMEM) have been demonstrated to play a role in cancer development and chemo-resistance. TMEM205 and its co-localized protein CD1B are expressed higher in platinum-resistant ovarian cancer cell lines and patient samples than in platinum-sensitive groups. TMEM205 regulates chemo-resistant associated proteins like exosome-regulatory proteins Rab11 and contributes resistance via increased exosomal efflux of intracellular platinum. Furthermore, they found TMEM205 inhibitors, L-2663, reduced exosome secretion, inhibited exosomal platinum efflux, and re-sensitized to platinum in resistant cells^47^.

Cleft lip and palate transmembrane protein 1-like (CLPTM1L)/cisplatin resistance-related protein 9 (CRR9) are cytoprotective oncofetal protein and chemo-resistance factors on the tumor cell surface. Many types of research have demonstrated that overexpression of CLPTM1L on the plasma membrane of ovarian cancer cells was associated with poor outcomes in serous ovarian adenocarcinoma. Except for the cell-autonomous effects of CLPTM1L on the resistance, they also found CLPTM1L from exosomes conferred chemo-resistance to bystander ovarian cancer cells in an ectodomain-dependent manner. Therefore, human anti-CLPTM1L mAbs could re-sensitize to platinum-resistant ovarian cancer cells achieved in orthotopic isografts and patient-derived cisplatin-resistant xenografts models^48^. So it's an important mechanism that increases exosome release and exosome-mediated cisplatin efflux in chemo-resistant ovarian cancer.

### Transfer chemotherapy resistance through drug exporters

Exosomes are formed by the inward budding of the multivesicular body membrane, which is usually controlled by the endosomal sorting complex required for transport (ESCRT). Finally, they are released outside the donor cells and fused with the plasma membrane of the recipient cells. Drug-resistant cells release exosomes and transfer their cargoes like P-glycoprotein (P-gp) to drug-sensitive cells, which contributes to disseminating therapy resistance phenotype in vivo and in vitro.

Levchenko et al. reported that P-gp transferred between tumor cells and tumor stroma reserved their functions and conferred drug resistance upon the recipient cells in 2004 for the first time. Furthermore, in solid metastatic tumors, they observed that the number of tumors expressing P-gp increased after chemotherapy^49^. On this basis, Bebawy et al. showed that microparticles (MPs) shed from drug-resistant tumor cells transferred functional P-gp to drug-sensitive recipient cells in vitro^50^. The research by Zhang FF et al. suggested that a drug-sensitive breast cancer cell line acquired a drug-resistant phenotype under co-culturing with exosomes extracted from its docetaxel-resistant counterpart. In addition, paclitaxel-resistant human ovarian cancer cells (A2780/PTX) released P-gp-contained exosomes to the extracellular space, which led to a redistribution of the chemotherapeutic drug in the wild-type parental line (A2780/WT) and increased five-times and more than five-times higher resistance to adriamycin and paclitaxel^51^.

### Transfer annexin A3 between cancer cells

Annexin A3 is a part of the phospholipid binding proteins family, which rely on calcium channels and participate in membrane fusion, cell exocytosis, modulation of ion channels, and signaling transduction^52^. It has been proved that the expression of annexin A3 is notably up-regulated in ovarian cancer cells, which are resistant to cisplatin, carboplatin, and platinum. Yan et al. found that annexin A3 selectively conferred resistance to the platinum by altering the transmembrane transportation of cisplatin and reducing their intracellular concentration, which led to a reduced p53 response to the drugs^53^. Moreover, Yin et al. found that Annexin A3 expressed higher in cisplatin-resistant ovarian cancer cell lines A2780/cis and SKOV3/cis, compared with their sensitive cells. Furthermore, serum levels of annexin A3 were higher in platinum-resistant patients, and it is proved that Annexin A3 upregulated in platinum-resistant cells by decreasing intracellular concentration of platinum and inhibiting cancer cell apoptosis. They observed that cancer cells expressing annexin A3 in high concentration released many exosomes, and exosomes serve as vehicles to transfer annexin A3 between cells to induce drug resistance^5^. These results suggested that the up-regulation of Annexin A3 played a critical role in increasing platinum resistance. It could be a biomarker for predicting sensitivity to platinum-based therapies in ovarian cancer patients.

### Mediate chemotherapy resistance by increasing DNMT1

Except for genetic factors, it has been proved that epigenetic changes like aberrant genomic DNA methylation and histone modifications are involved in tumor initiation, progress, recurrence, and drug resistance. DNA methyltransferase 1 (DNMT1) is essential to maintain genome‐wide methylation during DNA replication and damage repair. It has not been investigated that exosomes regulate epigenetic factors such as DNMT1 in ovarian cancer. Studies by Ercan et al. suggested that RGS10 was reduced via promoter hypermethylation mediated by HDAC1 or DNMT1 and supported that inhibition of HDAC1 or DNMT1 was viewed as an adjuvant therapeutic approach to overcome chemo-resistance in ovarian cancer^54^. The study by Cao et al. suggested that DNMT1 mRNA was packaged explicitly into exosomes for epigenetic signaling connection. Exosomes from the conditioned medium of tumor cells, in turn, promoted DNMT1's expression in host cells, which eventually led to cisplatin resistance. In a word, it could be an underlying mechanism that exosomes regulated the expression of DNMT1 and then contributed to resistance to cisplatin in ovarian cancer^39^.

### Regulate apoptosis

Apoptosis evasion is involved in tumor recurrence and chemotherapy resistance. Exosomes decrease pro-apoptotic signaling and increase anti-apoptotic signals to promote cell survival. Cisplatin dissociates GSN from the GSN-FLIP complex in chemo-sensitive cells, promoting GSN cleavage and caspase-3 activation. Werehene et al. reported for the first time the functions of exosomes containing plasma gelsolin (pGSN) (Ex-pGSN) on chemosensitivity in ovarian cancer. Ex-pGSN in chemo-resistant cells induces resistance in chemo-sensitive cells by targeting α5β1 integrin-FAK-Akt-HIF1α signaling pathway, upregulating pGSN, and inhibiting cisplatin-induced apoptosis^55^. Then they reported that chemo-resistant OC cells produced increased levels of exosomal pGSN that induced apoptosis of CD8+ T cell and reduced interferon-gamma (IFNγ) secretion, which supported that exosomal pGSN contributed to chemoresistance by immunosurveillance^56^. Guo et al. demonstrated that CAF-derived exosomes deliver miR-98-5p to promote cisplatin resistance in ovarian cancer by inhibiting CDKN1A, a key regulator for cell cycle arrest and apoptosis^57^.

### Promote EMT

Epithelial-to-Mesenchymal Transition is a complex phenomenon that involves tumor initiation and metastasis and increases resistance to chemotherapy and immunological therapy. Through EMT, epithelial cells in the tumor microenvironment lose their apical-basal polarity and become mesenchymal cells to promote cancer metastasis. In breast and ovarian cancer, invasiveness is an essential aggressive property. The first-line chemotherapy for HGSOC patients consists of platinum agents and paclitaxel. Several studies showed that tumor cells resistant to carboplatin and paclitaxel acquired a mesenchymal phenotype associated with EMT, which regarded EMT as the driver of chemotherapy resistance. In this review article, EMT-mediated mechanisms leading to therapy resistance in tumor cells include the following mechanisms: decreased cisplatin influx mediated by the copper transporter 1 (CRT1), an increased outflow of drugs, increased DNA damage repair, capacity by activating the PARP enzyme, inhibited p53-mediated cell apoptosis signals, changes in cell cycle, changes in different cellular pathways like TGFβ-SMAD, JAK/STAT. In another research, Bhattacharya et al. observed that CD44s, a mesenchymal spliced variant, was upregulated by TGF-β1-induced EMT. Furthermore, in ovarian cancer cells, overexpression of this mesenchymal isoform induced EMT, subsequently gaining stem-like characteristics and chemotherapy resistance^58^.

When platinum-sensitive cell A2780 was incubated with exosomes from platinum-resistant cells, it showed a two-fold increase in cell viability upon treatment groups treated with carboplatin compared to the control groups. Crow et al. discovered that previously unreported somatic mutations in the Mothers Against Decapentaplegic Homolog 4 (SMAD4) enhanced the chemo-resistance of epithelial ovarian cancer. SMAD4 is an essential part of TGF-β/SMAD signaling, and mutant SMAD4 ovarian cancer cells decrease PDCD4's expression, which regulates EMT. Also, they found that resistant A2780 cells, which exogenously expressed SMAD4 mutations, presented up-regulation of EMT markers and secreted exosomes, which increased by 1.7 fold in chemotherapeutic resistance compared to previously sensitive A2780 cells. Besides, it represented a novel mechanism that exosomes secreted from platinum-resistant ovarian cancer cells could continue the EMT phenotype and develop platinum-resistant cell subgroups^40^.

Cancer-derived fibroblasts are essential components in the tumor environment that interact with tumor cells to mediate progression, metastasis, and chemotherapy resistance. Li et al. presented that exosomes derived from cancer-associated fibroblasts were co-cultured with ovarian cells SKOV-3 and CAOV-3 induced malignant tumor behaviors, including an increased potential of migration and invasion and promoting EMT through activating the SMAD signaling pathway^59^. In addition, their results showed that TGF-β1 in CAF-derived exosomes propels ovarian cancer cells to a more aggressive phenotype, proving that it could be a potential treatment in ovarian cancer targeting CAF-derived exosomes. Hu et al. identified that CAFs directly transferred exosomes to colorectal cancer (CRC) cells to increase the secretion of miR-92a-3p. Elevated expression of miR-92a-3p directly inhibited FBXW7 and MOAP1 to activate the Wnt/β-catenin pathway and inhibited mitochondrial apoptosis, contributing to cell stemness, EMT, tumor metastasis, and drugs 5-FU resistance. In addition, they detected the level of exosomal miR-92a-3p in the serum of 18 cases. They showed that exosomal miR-92a-3p was increased in 5-FU-resistant VRC patients, indicating it might predict chemotherapy resistance in CRC patients^60^.

Increasing evidence has demonstrated that tumor cells can acquire chemo-resistance undergoing many tumor types. Exosomes may induce the characteristics of EMT cell morphology to induce chemotherapy resistance, indicating that targeting EMT is a feasible method for reversing resistance.

### Transfer miRNAs to sensitive cancer cells

miRNAs are short, non-coding RNAs 22 nucleotides in length, involved in post-transcription genetic modification. Interestingly, miRNAs encapsulated in exosomes are protected by exosomes from the degradation of RNases compared with naked miRNAs. It is worth noting that cells exposed to chemotherapeutic agents selectively parcel miRNAs into exosomes and are associated with chemotherapy resistance. Exosomes have been found to transfer miRNAs from drug-resistant cells to drug-sensitive ones, contributing to oncogenesis, the pre-metastatic niche formation, tumor migration, tumor invasion, and ultimately drug resistance by regulating drug-sensitive cells' expression locally and systemically in the tumor environment^61^, ^62^. In addition, there is reliable evidence that showed miRNAs transported by exosomes are used as a divinable biomarker of response to chemotherapy and a prognostic biomarker for developing chemotherapy resistance^63^. However, miRNAs in exosomes associated with ovarian cancer drug resistance have not been covered much, and it is challenging to identify canonical pathways in miRNA-mediated chemotherapeutic resistance.

Exosomes transfer miRNAs to sensitive tumor cells and induce chemotherapy resistance by inducing anti-apoptotic signals. Au Yeung et al. found that exosomes isolated from cancer-associated adipocytes and fibroblasts expressed a significantly higher level of miR-21 which could decrease apoptotic protease-activating factor 1 (APAF1) protein's expression in neighboring tumor cells and increase the chemo-resistance to paclitaxel^27^. Pink et al. presented that miR-21-3p in exosomes could increase resistance to cisplatin by suppressing protein-coding gene neuron navigator3's expression in the A2780 cells and cisplatin-resistant variant termed CP70 cells^41^. Additionally, several studies indicated that exosomal miR-21 was found to be elevated in advanced colorectal cancer and breast cancer.

Furthermore, exosomal miR-433 promoted resistance to paclitaxel by inducing cellular senescence and inhibiting the proliferation of neighboring cells^42^. They showed that stable miR-433 expression in ovarian cancer cells A2780 induced cellular senescence using morphological changes and downregulated phosphorylated retinoblastoma (p-Rb), ascribed to a miR-433-dependent downregulation of cyclin-dependent kinase6 (CDK6).

The abnormal tumor angiogenesis affects the delivery and sensitivity of chemotherapy drugs in tumor cells. So inhibiting tumor angiogenesis and inducing normalization decreases the vessel wall's permeability, thus increasing the accumulation of chemotherapeutics and improving drug sensitivity. Targeted delivery of miR-484 via RGD-modified exosomes inhibits tumor angiogenesis in ovarian cancer, which enhances the chemotherapy sensitivity and prolongs the survival time of tumor-bearing mice in the OC xenograft model after chemotherapy^64^.

DDP-Exos, exosomes derived from SKOV3/cisplatin (SKOV3/DDP) cells, were used to treat SKOV3 cells in vitro. The level of pyruvate dehydrogenase E1 subunit alpha 1 (PDHA1) in DDP-resistant SKOV3 cells was decreased significantly while miR-21-5p increased compared with control groups. They found that SKOV3/DDP-resistant-Exos treatment increased glycolysis, improved cell survival, and inhibited chemosensitivity of SKOV3 cells by upregulating PDHA1 exosomal miR-21-5p derived from DDP-resistant SKOV3 cells^65^. Although they demonstrated a new role of the exosomal-miR-21-5p/PDHA1 axis in chemosensitivity in SKOV3 OC cells, more in-vitro cell studies and vivo animal models should be carried out to verify the presented result.

Exosomal miR-429 secreted by multidrug-resistant SKOV3 cells displayed higher expression than sensitive A2780 cells. miR-429 dysregulation increased cell viability and chemo-resistance among lots of cancers. Exosomal miR-429 induced DDP resistance via targeting the calcium-sensing receptor (CASR)/STAT3 pathway in A2780 cells and xenograft mice tumors. So targeting exosomal miR-429 might be a novel therapeutic target in epithelial ovarian cancer^66^.

Zhu and colleagues also revealed that exosomal miR-223 derived from hypoxic macrophages could enhance drug resistance in epithelial ovarian cancer cells via the PTEN-PI3K/AKT pathways in vitro and in vivo. Moreover, exosomal miR-223 could be used as a biomarker for predicting response to chemotherapy and might be considered a possible drug target to overcome chemoresistance in advanced epithelial ovarian cancer patients^67^.

### Regulate transduction signals

The PI3K/AKT/ mTOR signaling pathway plays a prominent role in tumor progression, inhibiting apoptosis and resistance to chemotherapy^68^. Zhu et al. showed that exosomes derived from tumor-associated macrophage (TAM) transferred miR-233 to epithelial ovarian cancer cells to promote drug resistance under the mediation of the PTEN-PI3K/AKT signaling pathway^67^. Ip et al. studied the role of the PI3K/AKT/mTOR pathway in maintaining stemness and chemo-resistance in ovarian cancer stem cells. Treatment of SKOV3 stem cells with LY294002, the PI3K/AKT inhibitor, suppressed ABCG2's expression and P-gp, increasing sensitivity to chemotherapeutic drugs^69^. According to a new study, Li et al synthesized bioinspired hybrid nanoparticles named miR497/TP-HENPs that fused cRGD-modified liposomes and CD47-expressing tumor exosomes and encapsulated both chemotherapy agents triptolide (TP) and miR497. Furthermore, miR497/TP-HENPs overcome the chemoresistance by blocking OC's PI3K/AKT/mTOR signaling pathways 70. Compared with previous studies, their job helped achieve better clinical effects and settle the dilemma of chemotherapy resistance. Cisplatin treatment increases the release of exosomes, which influences the primary phenotype of neighboring naïve cells and improves cisplatin resistance via the P38 and JNK pathways when taken up by bystander cells^71^. These studies suggest that exosomes regulate signal pathways to change chemotherapy resistance.

## The detection of exosomes in clinical contexts

The most useful detection method in ovarian cancer patients is the concentration of serum carbohydr5ate antigen 125 (CA125) and transvaginal ultrasound (TVS). However, it had limitations at the early stage of ovarian cancer, so developing higher specific and sensitive biomarkers is attractive to improve the diagnostic rate of ovarian cancer patients. In recent years, liquid biopsy has become a promising new area of early detection of ovarian cancer, which has the advantage of being less invasive, easier to obtain, and easier for serial measurements in the period of treatment. It includes the collection and analysis of circulating tumor cells (CTCs), circulating tumor DNA (ctDNA), circulating cell-free miRNAs (cfmiRNAs), and circulating exosomes^72^. It is ideal to regard exosomes as biomarkers for cancer diagnosis and the prediction and monitoring of therapeutic response because they have many advantages. First, exosomes are accessible in nearly all bodily fluids, such as blood, saliva, urine, breast milk, and ascites. Secondly, they have an advantage in high circulation stability because of a lipid bilayer in the exosome membrane. Thirdly, the enriched contents within exosomes are reservoirs of proteins, mRNAs, and miRNAs reflecting the current state of the tumor, and these contents can be collected in a non-invasive way. Above all this made exosomes an ideal diagnostic and prognostic biomarkers. Furthermore, traditional exosome isolation and detection methods are time-consuming, expensive, and complex, so they could not be used in clinical testing. Further development and standardization of the technologies and methods for exosomes isolation, analysis, specific inhibition, and removal from blood circulation as well as an in-depth understanding of the molecular regulations of exosomes biogenesis and signal transduction are essential for exosomes to serve as important tools in advanced ovarian cancer patient's prognosis and survival period.

Recent studies have demonstrated that exosome miRNAs have the potential to serve as biomarkers for the diagnosis and prognosis of OC. The expression levels of 5 miRNAs (miR-205-5p, miR-145-5p, miR-10a-5p, miR-346, and miR-328-3p) in plasma exosomes of patients with OC significantly elevated, which indicated that the expression levels of exosomal miRNAs might be considered as noninvasive biomarkers for the diagnosis of OC patients 73. The exosomal transfer of miR-21 could promote oncogenic transformation in target cells distant from the primary tumor and be used as a diagnostic tool. We found two clinical trials led by the same team in China, NCT03738319, and NCT03742856, aiming to analyze the expression of exosomal miRNA and long non-coding RNA (lncRNA) by next-generation sequencing in patients with HGSOC and benign gynecologic diseases. The candidate miRNA/lncRNA will be validated as a biomarker for the detection and prognosis of HGSOC. However, the status of these two trials is currently listed as “unknown”. In current years, the commonly used method to detect miRNA in exosomes is exosome isolation, miRNA extraction, cDNA synthesis, and real-time PCR analysis. However, this method is time-consuming, laborious, and expensive. Future jobs to develop exosomal miRNAs for diagnosis of EOC at the early stage included standardized high-throughput exosomal miRNA isolation and detection, consensus protocols on diagnosing EOC, and prediction models of exosomal miRNAs.

Except for miRNAs, exosomal proteins might also be potential biomarkers for the diagnosis of OC. It is also demonstrated that the serum levels of HSP27 were significantly increased in patients with epithelial ovarian cancer, which was secreted by exosomes. Therefore, serum HSP27 may be used as a potential biomarker of response to treatment in epithelial ovarian cancer patients^74^. CD24 could be found in the cytoplasm inside MVBs and released into the extracellular environment via exosomes, it is correlated with more aggressive forms of ovarian carcinoma so it worse the prognoses and shorten patients' survival times. The increase of epithelial cell adhesion molecule (EpCAM) concentration is associated with the stages of ovarian cancer, which indicates EpCAM in exosomes could be useful for the diagnosis of ovarian cancer. So EpCAM and CD24 could be useful a marker for the diagnosis of ovarian cancer patients and the detection of TD-exosome^75^. However, even if several literatures seem promising, there remain no findings aiming at evaluating the role of the mentioned exosome biomarkers in the clinic. Future research is certainly represented by solving these issues in the clinical aspect and seeking the most reliable exosome biomarkers.

## Therapeutic potential of exosomes in ovarian cancer

**Figure 4:**
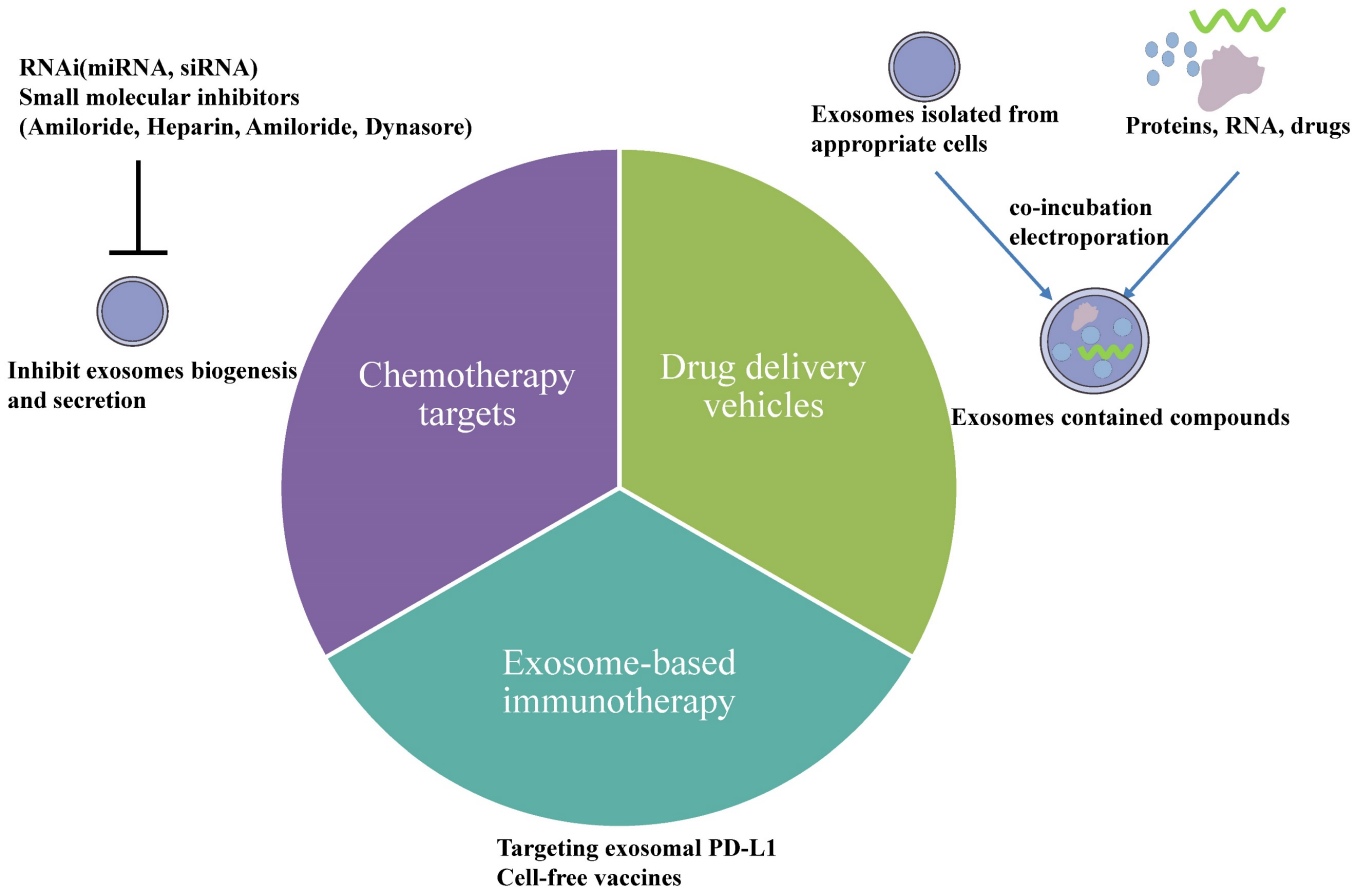
Therapeutic potential of exosomes in ovarian cancer. 1) Drug delivery vehicles. Nanoscale drug delivery systems electroporate chemotherapeutic drugs and nucleic acids into exosomes collected from an appropriate cell culture or co-incubate them. Once the exosomes are successfully loaded, they could be used for downstream therapeutic applications. 2) Chemotherapy targets. Inhibiting exosome biogenesis and secretion is one particular therapeutic strategy for overcoming resistance either by using RNAi or other small molecular inhibitors. 3) Exosome-based immunotherapy. Exosomal PD-L1 could suppress T-cell activation, indicating that blocking specific targets on exosomal PD-L1 using anti-PD-L1 antibodies could improve tumor immunotherapy responsiveness. In addition, exosomes could be used as cell-free vaccines in cancer immunotherapy because they contain numerous tumor antigens.

In recent years, much evidence has displayed that exosomes have been used as therapeutic targets for cancer. Exosomes released from a cell line are heterogeneous, meaning treatment is efficient for one type of ovarian cancer patient but not for another. Therefore, targeted and personalized treatments have obtained more and more attention in current searches. Exosomes are ideal therapeutic tools for advancing ovarian cancer patients. Many strategies exist to reverse the chemotherapy resistance by exosomes in ovarian cancer, including utilizing exosomes as delivery vehicles which can increase drugs' concentration and block exosome secretion from ovarian cancer cells. Furthermore, exosomes play an essential role in anti-tumor immune responses, and they are specific biomarkers to predict the efficiency of immune responses. Nowadays, there exist three treatments based on exosomes (Fig. 4): 1. drug delivery vehicles, 2. treatment targets, and 3. immunotherapeutic approaches.

### Exosomes as drug delivery vehicles

A suitable drug delivery vehicle has been in great demand for cancer treatment because it improves the therapeutic efficacy and safety profile of the chemotherapeutic drugs and their metabolites. Exosomes are used as drug-delivered vehicles because of their high stability in circulation, high permeability, and low immunogenicity. They deliver drugs and their metabolizes across the biological membrane into the cytoplasm of recipient cells without the endosomal and lysosomal pathways. Nanoscale drug delivery systems incubate or electroporate chemotherapeutic drugs and nucleic acids into exosomes presenting more opportunities for unsolved clinical issues like chemotherapeutic resistance^76^. For example, Kim et al. showed that incorporating paclitaxel into exosomes increased solubility and cytotoxicity more than fifty times in multidrug-resistant (MDR) cell lines MDCK_MDR1_ to overcome P-gp-mediated drug efflux^77^. Another study showed that bioinspired hybrid nanoparticles called miR-497/TP-HENPs, formed by CD47-expressed exosomes from SKOV3-CDDP cells and cRGD-modified liposomes, increased drug delivery to target tumor sites by evading MPS clearance. It could be considered an effective cancer therapy to overcome chemo-resistance^70^.

Although many studies have demonstrated that exosomes assist cancer treatment, studying exosome application and nanotechnology development would require more clinical research. A phase I trial study (NCT03608631) studied the best dose and side effects of mesenchymal stromal cells-derived exosomes with KrasG12D siRNA in treating metastatic pancreatic cancer patients with KrasG12D mutation, aiming to treat pancreatic cancer by exosomes. In addition, a Phase II clinical trial (NCT01854866) recruited thirty malignant ascites or pleural effusion patients and they were injected with tumor cell-derived microparticles packaging chemotherapeutic drugs. It demonstrated that tumor cell-derived microparticles might be useful for treating malignant ascites and pleural effusion. However, there remains to be an urgent problem dealing with the isolation, characterization, storage, and administration of exosomes, to achieve mass production of clinical therapeutic exosomes. Furthermore, it remains to be answered which cell type to utilize for the isolation and purification of exosomes.

### Exosomes as chemotherapy targets

Considering the importance of exosomes in chemotherapy resistance, it has a clinical implication for prospective tumor therapies to remove tumor-derived exosomes through targeting exosome biogenesis and secretion. The levels of exosomes are down-regulated by using RNAi and other small molecular inhibitors in exosome biogenesis and secretion. Under hypoxic conditions, STAT3 knockdown changed the expression of Rab27a and Rab7 to reduce exosome release. Combined inhibitor Amiloride, a STAT3 inhibitor with cisplatin, blocked the release of exosomes and significantly inhibited colony formation numbers and cell proliferation, demonstrating that STAT3 inhibitor increased chemosensitivity to cisplatin^45^. Samuel et al. suggested blocking EV transfer in vitro sensitized ovarian cancer cells to cisplatin using EV uptake inhibitors, including heparin, amiloride, and dynasore^71^. However, it demands many in-vivo studies on blocking exosome transfer between cancer cells to increase chemotherapy sensitivity. miR-233 delivered via exosomes derived from TAM promoted drug resistance in ovarian cancer. It might be a novel treatment approach to regulate exosome-derived miRNA released by macrophages in the tumor microenvironment^67^. Studies by Au Yeung suggested that miR-21 in exosomes developed a malignant phenotype and promoted ovarian cancer paclitaxel resistance. It would be an alternative method to inhibit the transfer of exosome-derived miR-21 to treat metastatic and recurrent ovarian cancer^27^. Also, exosome Annexin A3 might be a prognostic biomarker of resistance to platinum chemotherapy in EOC patients with high sensitivity and specificity. In conclusion, these studies proved that targeted inactivation or elimination of exosomes would provide new insights into cancer treatment.

### Exosomes in future immunotherapy

In recent years, several interventions, including immune checkpoint blockade, cancer vaccines, and adoptive cell therapy, have attracted interest among solid tumors like melanoma, NSCLC, and classical Hodgkin lymphoma. However, no approved immune treatments for ovarian cancer^78^. Several phases of clinical research revealed that immune checkpoint programmed death 1/PD-L1 inhibitors in ovarian cancer had shown a modest response rate^79^, ^80^. Therefore, it remains to be solved by optimizing immunotherapy treatment strategies and exploring more significant biomarkers for predicting immunotherapeutic response in ovarian cancer.

Notably, exosomes could be used as carriers for tumor immunotherapy because of their immunogenicity and transfer function. Also, they were involved in antigen presentation, activation of T cells, and trigger of CD8+T cell-dependent anti-tumor responses in vitro and in vivo. It's thought that PD-L1 generally interacts with PD-1 on the surface of tumor-infiltrating lymphocytes, resulting inhibition of T-cell signaling. In addition, PD-L1 also exists on the surface of exosomes. Exosomal PD-L1 suppresses CD8+ T cell function, including proliferation, cytotoxicity, and cytokine production in vitro and in vivo. So it represents an expected therapeutic method that genetically blocks exosome biogenesis. The release of exosomal PD-L1 can promote T cell activation and proliferation and overcome resistance to the current antibody approach^81^. The expression of tumor-derived exosomal PD-L1 is increased by IFN-γ secreted from CD8+ T lymphocytes. Furthermore, tumor cells secreted PD-L1 positive exosomes in the tumor microenvironment or to distant places by lymph nodes and targeted effector T cells to inhibit anti-tumor immunity^82^. So it's obvious that blocking specific targets on exosomal PD-L1 using anti-PD-L1 antibodies could improve tumor immunotherapy responsiveness. It's feasible that circulating exosomal PD-L1 is applied to a potential biomarker of tumor progression and response to immunotherapy. However, it remains a problem to be solved to detect circulating exosomal PD-L1 in ovarian cancer patients.

Recent research has shown that tumor-derived exosomes increase anti-tumor immune responses by inducing immune effector cells and suppressing the immune suppressor cells' response. Exosomes derived from natural killer cells(NK-Exo) could enhance ovarian cancer cells' killing effect in vitro by increasing DDP uptake and reversing the immunosuppression capacity of NK cells^83^. However, exosomes could induce apoptosis of activated CD8+T cells to play an immunosuppressive effect. Even so, exosome-based strategies for cancer immunotherapy could still be considered.

In addition, exosomes could be used as cell-free vaccines in cancer immunotherapy because they contain numerous tumor antigens. Cho et al. showed that exosomes derived from tumor cells were novel vesicles for delivering tumor-derived antigens to induce effective immune responses against tumors because of their immunogenicity^84^. Dendritic cells derived exosomes can more effectively stimulate CD4+ helper T cells and CD8+ CLTs proliferation and induce an anti-tumor immune response, representing a novel immunotherapy method for ovarian cancer patients^85^. Phase II Trial (NCT01159288) used a vaccination involving metronomic cyclophosphamide (mCTX) based on tumor antigen-loaded dendritic cell-derived exosomes (Dex) to treat unresectable non-small cell lung cancer patients. Although the Phase I trial could not monitor the induction of T cells after using Dex vaccines in patients, they validated a new process for the isolation of Dex with improved immune stimulatory capacities to treat 47 advanced unresectable NSCLC patients. Furthermore, vaccination within tumor-associated exosomes loaded T-cells could counteract CD4 + CD25 + Treg cell-mediated immunosuppression and trigger CTL long-term memory to stimulate immune responses^86^.

Therefore, how exosomes could deliver immunotherapy drugs and antigens to stimulate anti-tumor immune responses in the tumor microenvironment is vital regarding cancer treatment. Although exosomes have attracted more and more attention as cancer vaccines and carriers for various cancers, there have been no animal and clinical trials to verify their efficacy in ovarian cancer.

## Conclusion and future perspectives

Ovarian cancer threatens female gynecological health due to a high mortality rate. In recent years, new ideas about eliminating specific exosomes, encapsulating chemotherapeutic drugs in exosomes to protect against degradation, and inhibiting exosome biogenesis and secretion, have been proven to slow down tumor progression and improve chemotherapeutic sensitization. Current studies suggested that exosomes derived from tumor cells and peripheral body fluid were expected to use as predictable biomarkers for early diagnosis of ovarian cancer and treatment targets to overcome chemo-resistance. With their high stability in circulation and accessibility to obtain from the body fluid, detecting exosomes in the peripheral blood via liquid biopsies might be a promising method. However, the immaturity of the purification and detection methods hinders their translation into clinical application. We propose that larger multi-institutional studies including more pre-clinical and clinical research, standardization, and best strategies for exosome isolation and application remain implemented to move this field forward for the benefit of patients in clinical.

Exosomes could transfer multiple biological molecules to the recipient cells or the cancer microenvironment, so they might be served as a drug delivery system to overcome drug resistance. In addition, engineered exosomes achieve cell-specificity and tumor-specificity by modifying the surface molecules on exosomes. In the future, more studies are urged to investigate the utility of exosomes as natural delivery vehicles to guarantee the safety and accuracy of ovarian cancer patients' treatment at lower drug doses and fewer side effects.

In conclusion, although many studies on exosomes have confirmed their great diagnostic and therapeutic potential in improving the prognosis of ovarian cancer, challenges in this field need to be overcome before providing exosomes as biomarkers and treatment targets for ovarian cancer patients.

## Figures and Tables

**Table 1:** miRNAs derived from exosomes associated with chemoresistance in ovarian cancer

miRNAs in ovarian cancer	Pathways	The role of miRNAs in promoting chemoresistance in ovarian cancer.	Reference
miR-21	Apoptosis associated pathways	Bind with the APAF1 target to induce paclitaxel resistance in OVCA432 and SKOV3 cells.	27
miR-21-3p		Expression of miR-21-3p was 50-fold higher level in the A2780 cells and CP70 cells by suppressing the expression of NAV3.	41
miR-433		Induce cellular senescence by downregulating cyclin-dependent kinase6.	42
miR-484	Angiogenesis	Targeting RGD-modified exosomes inhibits tumor angiogenesis and increases sensitivity.	64
miR-21-5p	Increased glycolysis by miR-21-5p/PDHA1 axis	Increased glycolysis by upregulating PDHA1 exosomal miR-21-5p derived from DDP-resistant SKOV3 cells.	65
miR-429	Targeting CASR/STAT3 pathway	Induced DDP resistance targeting the CASR/STAT3 pathway in A2780 cells and xenograft mice tumors	66
miR-223	Targeting PTEN-PI3K/AKT pathways	Exosomal miR-223 derived from macrophages promoted drug resistance via the PTEN-PI3K/AKT pathway in SKOV3 cells and SKOV3 xenograft models.	67

## Abbreviations

EMT: Epithelial-to-Mesenchymal transition
MVBs: multivesicular bodies
NEM: N-ethylmaleimide
SNARE: soluble N-ethylmaleimide-sensitive factor attachment protein receptor
ILVs: intraluminal vesicles
MHC: major histocompatibility complex
CAFs: cancer-associated fibroblasts
TGFβ1: transforming growth factor 1
SMAD: somatic mutations in the Mothers Against Decapentaplegic Homolog
TSAP6: Tumor suppressed activated pathway6
VEGF: vascular endothelial growth factor
TNF-α: tumor necrosis factor α
MALAT1: metastasis-associated lung adenocarcinoma transcript 1
HUVECs: human umbilical vein endothelial cells
APCs: antigen-presenting cells
TAA: tumor-associated antigen
HR: homologous recombination
EOC: epithelial ovarian cancer
BRCA1/2: breast cancer susceptibility gene
HGSOC: high-grade serous ovarian cancer
PARP: Poly (ADP-ribose) polymerases
PFS: progression-free survival
CAAs: cancer-associated adipocytes
ALDH1: aldehyde-dehydrogenase 1
ABC: ATP binding Cassette
SP: side populations
NER: nucleotide excision repair
ERCC1: Excision Repair Cross-Complementing 1
NHEJ: Non-homologous end joining
DSB: DNA double-strand break
PARG: PAR glycohydrolase
PTIP: Pax2 transactivation domain-interacting protein
MRN: MRE11 nuclease
LAMP: lysosome-associated proteins
OGT: O-GlcNAcylation transferase
TMEM: Transmembrane family proteins
CLPTM1L: Cleft lip and palate transmembrane protein 1-like
CRR9: cisplatin resistance-related protein 9
ESCRT: endosomal sorting complex required for transport
P-gp: P-glycoprotein
DNMT1: DNA methyltransferase 1
Ex-pGSN: exosomes containing plasma gelsolin
IFNγ: interferon gamma
CRT1: copper transporter 1
SMAD4: somatic mutations in the Mothers Against Decapentaplegic Homolog 4
CRC: colorectal cancer
APAF1: apoptotic protease-activating factor 1
p-Rb: phosphorylated retinoblastoma
CDK6: cyclin-dependent kinase6
PDHA1: pyruvate dehydrogenase E1 subunit alpha 1
CASR: calcium-sensing receptor
TAM: tumor-associated macrophage
TP: triptolide
CA125: carbohydr5ate antigen 125
TVS: transvaginal ultrasound
CTCs: circulating tumor cells
ctDNA: circulating tumor DNA
cfmiRNAs: circulating cell-free miRNAs
lncRNA: long noncoding RNAs
EpCAM: epithelial cell adhesion molecule
MDR: multiple drug resistance
NK cell: natural killer cell
